# CD8^+^HLADR^+^ Regulatory T Cells Change With Aging: They Increase in Number, but Lose Checkpoint Inhibitory Molecules and Suppressive Function

**DOI:** 10.3389/fimmu.2018.01201

**Published:** 2018-06-04

**Authors:** Stella Lukas Yani, Michael Keller, Franz Leonard Melzer, Birgit Weinberger, Luca Pangrazzi, Sieghart Sopper, Klemens Trieb, Monia Lobina, Valeria Orrù, Edoardo Fiorillo, Francesco Cucca, Beatrix Grubeck-Loebenstein

**Affiliations:** ^1^Department of Immunology, Institute for Biomedical Aging Research, University of Innsbruck, Innsbruck, Austria; ^2^Clinic for Haematology and Oncology, Tyrolean Cancer Research Institute, Medical University of Innsbruck, Innsbruck, Austria; ^3^Department of Orthopedic Surgery, Hospital Wels-Grieskirchen, Wels, Austria; ^4^Istituto di Ricerca Genetica e Biomedica (IRGB), Consiglio Nazionale delle Ricerche (CNR), Monserrato, Italy

**Keywords:** CD8^+^ T cells, CD8^+^human leukocyte antigen–antigen D related^+^, aging, checkpoint inhibitory molecules, regulatory T cells

## Abstract

CD4^+^ regulatory T cells have been intensively studied during aging, but little is still known about age-related changes of other regulatory T cell subsets. It was, therefore, the goal of the present study to analyze CD8^+^human leukocyte antigen–antigen D related (HLADR)^+^ T cells in old age, a cell population reported to have suppressive activity and to be connected to specific genetic variants. We demonstrate a strong increase in the number of CD8^+^HLADR^+^ T cells with age in a cohort of female Sardinians as well as in elderly male and female persons from Austria. We also show that CD8^+^HLADR^+^ T cells lack classical activation molecules, such as CD69 and CD25, but contain increased numbers of checkpoint inhibitory molecules, such as cytotoxic T lymphocyte-associated antigen 4, T cell immunoglobulin and mucin protein-3, LAG-3, and PD-1, when compared with their HLADR^−^ counterparts. They also have the capacity to inhibit the proliferation of autologous peripheral blood mononuclear cells. This suppressive activity is, however, decreased when CD8^+^HLADR^+^ T cells from elderly persons are analyzed. In accordance with this finding, CD8^+^HLADR^+^ T cells from persons of old age contain lower percentages of checkpoint inhibitory molecules than young controls. We conclude that in spite of high abundance of a CD8^+^ regulatory T cell subset in old age its expression of checkpoint inhibitory molecules and its suppressive function on a per cell basis are reduced. Reduction of suppressive capacity may support uncontrolled subclinical inflammatory processes referred to as “inflamm-aging.”

## Introduction

We have recently reported genetic contributions to quantitative levels of 95 immune cell types encompassing 272 immune traits in a cohort of 1,629 individuals from four clustered Sardinian villages (1). One of these 95 cell types are human leukocyte antigen–antigen D related (HLADR)^+^ T cells. In the past, the expression of HLADR on human T cells has mainly been regarded as a marker of activated T cells (2–4). However, HLADR expression on CD4^+^ regulatory T cells (Tregs) has also been interpreted as a marker of a functionally distinct population of mature Tregs (5). Recently, HLADR expression on CD8^+^ T cells has been suggested to represent a marker of a natural human CD8^+^ regulatory T cell subset (6). This subset was shown to suppress the proliferation of autologous peripheral blood mononuclear cells (PBMCs) presumably by cell-to-cell contact with cytotoxic T-lymphocyte-associated antigen 4 (CTLA-4) signaling playing an essential role in this process. In the past, CD8^+^ T cells were the first to be described to have immunosuppressive properties (7, 8). However, difficulties in characterizing these “suppressor” CD8^+^ T cells and lack of specific markers delimited this area noticeably. Only when CD4^+^CD25^+^FOXP3^+^ T cells were shown to be strongly immunosuppressive (9–13), a global interest in the downregulation of immune activity by T cells re-emerged. Apart from the description of many interesting specific properties, CD4^+^ Tregs were shown to increase in number in some important conditions, such as in patients with tumors (14) or during aging [reviewed in Ref. (15)]. In the latter context it was of interest that some Treg populations, specifically naturally occurring Tregs, seemed to accumulate in old age, whereas inducible Tregs decrease in elderly persons. Little information is, however, still available on the functional competence of CD4^+^ Tregs in old age. The worldwide strong interest in CD4 Tregs also stimulated new research efforts in the analysis of potential CD8^+^ suppressor cells. Different subsets were defined, such as CD8^+^CD28 low cells (16–20), CD8^+^CD122^+^ cells in mice (21–23), CD8^+^C-X-C motif chemokine receptor 3^+^ cells in humans (24) and CD8^+^CD39^+^FOXP3^+^ cells (25). CD8^+^HLADR^+^ T cells were therefore just one of several CD8^+^ T cell subsets believed to have regulatory properties (6). In view of the clear genetic regulation of this population exerted by a genetic variant in the *CIITA* gene region (1) and the fact that the composition of the CD8^+^ population characteristically changes with age (26), we became interested in elucidating potential age-related changes in the number and function of CD8^+^HLADR^+^ T cells. We now demonstrate that CD8^+^HLADR^+^ T cells increase in number with aging, but lose suppressive activity on a per cell basis. This may challenge the homeostatic balance between immune cell sub-populations in old age and support the development of inflammation.

## Materials and Methods

### Study Subjects

Samples from three different cohorts were used for this study. Details regarding the probands’ characteristics are summarized in Table 1.

**Table 1:** Demographic data on the cohorts used.

Cohort	Material	*n*	*n* Female	Age (median)	Age (range)	Site of recruitment
A	Peripheral blood (PB)	91	91 (100%)	49 years	18–89 years	Lanusei, Sardinia, Italy
B	PB	26	16 (61%)	26 years	21–29 years	Innsbruck, Austria
B	PB	22	13 (59%)	77 years	71–88 years	Innsbruck, Austria
C	Bone marrow	29	17 (58%)	68 years	39–87 years	Wels, Austria
C	PB	29	17 (58%)	68 years	39–87 years	Wels, Austria

#### Cohort A

Peripheral blood (PB) samples were obtained from 91 healthy females recruited in Lanusei, Sardinia, Italy. The exclusion criteria were the following:

Persons who experienced significant variation of body temperature or received vaccination in the 2 weeks before the blood draw, as well as persons under antimicrobial treatment were excluded from the study. Persons with pathological conditions were also excluded.

#### Cohort B

Peripheral blood samples were obtained from healthy Austrian individuals who did not receive immunomodulatory drugs or suffer from diseases known to influence the immune system, such as autoimmune diseases and cancer. None of them was frail or had symptoms of cognitive impairment.

#### Cohort C

Bone marrow (BM) and PB-paired samples were obtained from patients who underwent hip replacement surgery in Wels, Austria. The patients did not receive immunomodulatory drugs or suffer from diseases known to influence the immune system, such as autoimmune diseases and cancer.

### Mononuclear Cell Isolation

Peripheral blood mononuclear cells from heparinized blood were purified by Ficoll-Hypaque density gradient centrifugation (GE Healthcare Life Sciences). Cells were washed with complete RPMI medium (RPMI 1640 supplemented with 10% FCS, 100 U/ml penicillin, and 100 µg/ml streptomycin; Invitrogen). PBMCs from cohort A were frozen according to a standard protocol using 10% DMSO as cryoprotective agent. PBMCs from Cohort B and C were freshly used.

Bone marrow samples (cohort C) were obtained from the femur shaft of patients of varying age (Table 1) during hip replacement surgery. A biopsy of *Substantia spongiosa ossium*, which would otherwise have been discarded, was used to isolate BM mononuclear cells (BMMCs). BM biopsies were fragmented, washed once with complete RPMI medium (RPMI 1640 supplemented with 10% FCS, 100 U/ml penicillin, and 100 µg/ml streptomycin; Invitrogen), and treated with purified collagenase (CLSPA, Worthington Biochemical; 20 U/ml in complete RPMI medium) for 1 h at 37°C. BM biopsies were then centrifuged and BMMCs purified by density gradient centrifugation (Ficoll-Hypaque). BMMCs were freshly used.

### Cell Sorting

T cells were isolated from fresh PBMCs by magnetic cell sorting using the MACS human Pan T cell isolation kit (Miltenyi Biotech) following manufacturer’s protocol. Purified T cells were then stained with anti-CD8 PerCP (RPA-T8), anti-CD28 BV421 (L293), anti-HLADR PeCy7 (G46-6) Abs, all from BD Biosciences. Labeled cells were sorted with a FACSAria II flow cytometer (BD Biosciences). Cells were either sorted into CD8^+^HLADR^+^, CD8^+^HLADR^−^ or into CD8^+^CD28^+^HLADR^+^, CD8^+^CD28^+^HLADR^−^, CD8^+^CD28^-^HLADR^+^ or CD8^+^CD28^-^HLADR^−^ T cells. Fluorescence minus one (FMO) was used as control. The cells were collected into RPMI 1640 medium containing 15% FCS and washed once prior to further studies. The purity of each subset was >95% as determined by flow cytometry.

### Flow Cytometric Analyses

Peripheral blood mononuclear cells and BMMCs were stained with anti-CD4 VioGreen (REA623), anti-CD3 Vio770 (REA613) from Miltenyi Biotech, anti-CD45RA PerCp (HI100), anti-CD28 APC (28.2), anti-CTLA-4 PE (BNI3), anti-T cell immunoglobulin and mucin protein-3 (TIM-3) PerCP (F38-2E2), anti-programmed death 1 (PD-1) FITC (EH12.2H7), anti-lymphocyte activation gene-3 (LAG-3) APC (7H2C65) from Biolegend, anti-HLADR PeCy7 (G46-6), anti-CD28 BV421 (28.2), anti-CD25 APC (2A3), and anti-CD69 PE (FN 50) from BD Biosciences. Intracellular detection of FOXP3 with anti-FOXP3 FITC Abs was performed using fixed and permeabilized cells following the manufacturer’s instructions. FMO was used as control. Dead cells were excluded by forward and side scatter characteristics and by using either 7-AAD or fixable viability dye Zombie violet (Biolegend).

For each sample at least 2 × 10^6^ total events were acquired using a FACScanto II and FACSSymphony (BD Biosciences). The data were analyzed using FlowJo software.

### Suppression Assay

The capacity of sorted CD8^+^HLADR^+^, CD8^+^HLADR^−^, CD8^+^CD28^+^HLADR^+^, CD8^+^CD28^+^HLADR^−^, CD8^+^CD28^-^HLADR^+^, and of CD8^+^CD28^−^HLADR^−^ T cells to suppress the proliferation of responder autologous PBMCs was analyzed by CFSE dilution.

CFSE dilution method: responder autologous PBMCs (1 × 10^5^) labeled with 1 µM CFSE (Invitrogen) were cultured with highly purified unlabeled CD8^+^HLADR^+^, CD8^+^HLADR^−^, CD8^+^CD28^+^HLADR^+^, CD8^+^CD28^+^HLADR^−^, CD8^+^CD28^−^HLADR^+^, and CD8^+^CD28^−^HLADR^−^ T cells at different responder: suppressor cell ratios (2:1, 4:1, and 8:1). Cells were stimulated with 1 µg/ml anti-CD3 (BD Pharmingen) and 0.5 µg/ml anti-CD28 (BD Pharmingen) Abs in 96-well round-bottom plate and cultured in complete medium. After 4 days of culture cells were stained with anti-CD4 BV 510 (BD Pharmingen), anti-CD8 PeCy7 (BD Pharmingen) and proliferation of CFSE-labeled cells was assessed by flow cytometry. Percentage of suppression was calculated as 100 minus the percentage of responder PBMCs, which underwent one or more cell divisions (proliferated PBMCs) in the presence of suppressor cells divided by the percentage of proliferated PBMCs when cultured alone, and multiplied by 100 as shown in the following formula:

$$\text{\% suppression=100}-\frac{\left(\begin{array}{l} \text{proliferated PBMCs cultured}\\ \text{ with suppressor cells} \end{array}\right)}{\text{proliferated PBMCs cultured alone}}\times 100$$

The following controls for the suppression assay were used:

– CFSE-unlabeled and unstimulated (in the absence of stimulatory antibodies) cells,
– CFSE-labeled and unstimulated cells,
– CFSE-labeled and stimulated cells in the absence of suppressor cells.

### Neutralization Assay

To test the involvement of the checkpoint inhibitory molecules in the suppression mediated by CD8^+^HLADR^+^CD28^+^ T cells, neutralizing Abs against CTLA-4 (BD Biosciences), TIM-3 (Invitrogen), LAG-3 (Adipogen Life Sciences), and PD-1 (Invitrogen) were added to the co-cultures. All neutralization assays were performed under the culture conditions described above. The corresponding isotype-matched mAb IgG1 (Biozym) was used as control. Optimal neutralizing Ab concentrations were determined in pilot experiments.

### Statistical Analysis

Statistical significance was assessed by Spearman correlation analysis, Mann–Whitney *U*-test and Wilcoxon matched pairs test. *p-*Values below 0.05 were considered as significant.

### Study Approval

#### Cohort A

The study was approved by the Research Ethics and Bioethics Committee of the Consiglio Nazionale delle Ricerche (Italy). Written informed consent was received from participants prior to their inclusion in the study.

#### Cohort B

The study was approved by the Ethics Committees of the Medical University of Innsbruck (Austria). Written informed consent was received from participants prior to their inclusion in the study.

#### Cohort C

The study was approved by the Ethics Committees of the “Klinikum Wels-Grieskirchen” (Austria). Written informed consent was received from participants prior to their inclusion in the study.

## Results

### CD8^+^HLADR^+^ T Cells Increase With Age

**Figure 1:**
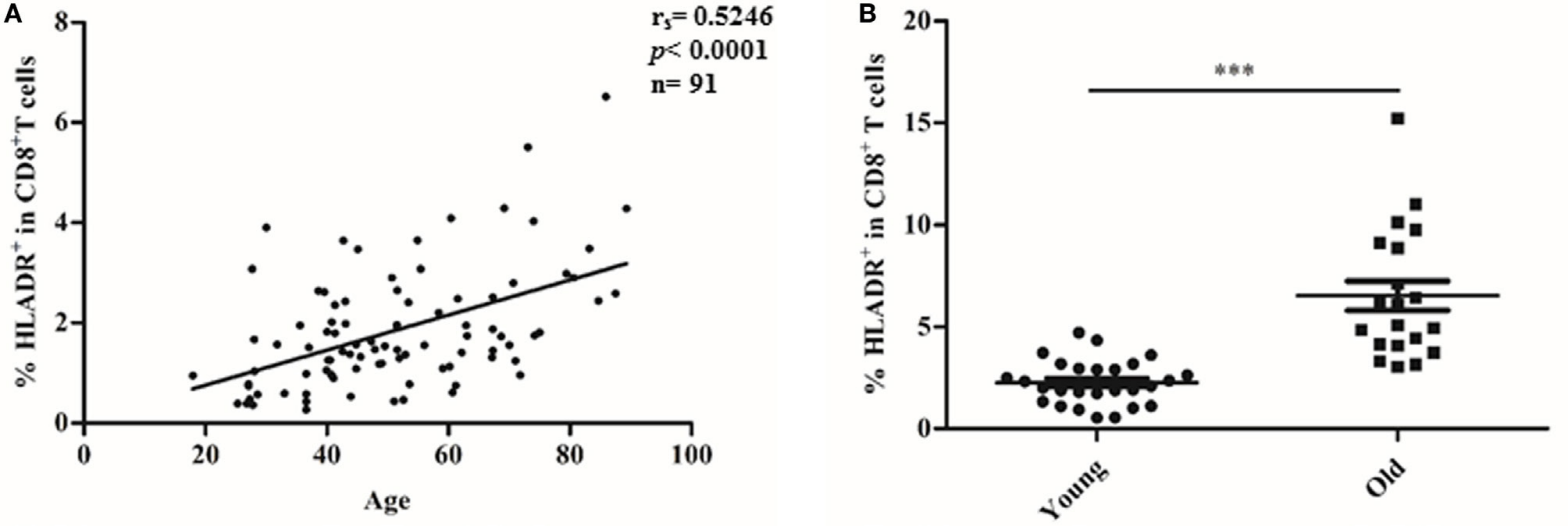
CD8^+^human leukocyte antigen–antigen D related (HLADR)^+^ T cells increase with age. HLADR^+^ cells (%) within CD3^+^CD8^+^ (100%) were measured by FACS analysis. **(A)** Correlation of CD8^+^HLADR^+^ cells in 91 female donors from Sardinia with age. Relationship between CD8^+^HLADR^+^ T cells (%) and age was assessed by Spearman correlation analysis; (*r*_s_), *p* value, and sample size (*n*) are indicated in **(B)** CD8^+^HLADR^+^ T cells in young vs elderly donors from Austria. Mean ± SEM are indicated for each group; young (<30 years), *n* = 26, old (≥70 years), *n* = 20. Statistical significance was assessed by Mann–Whitney test, ***p < 0.0001.

**Figure 2:**
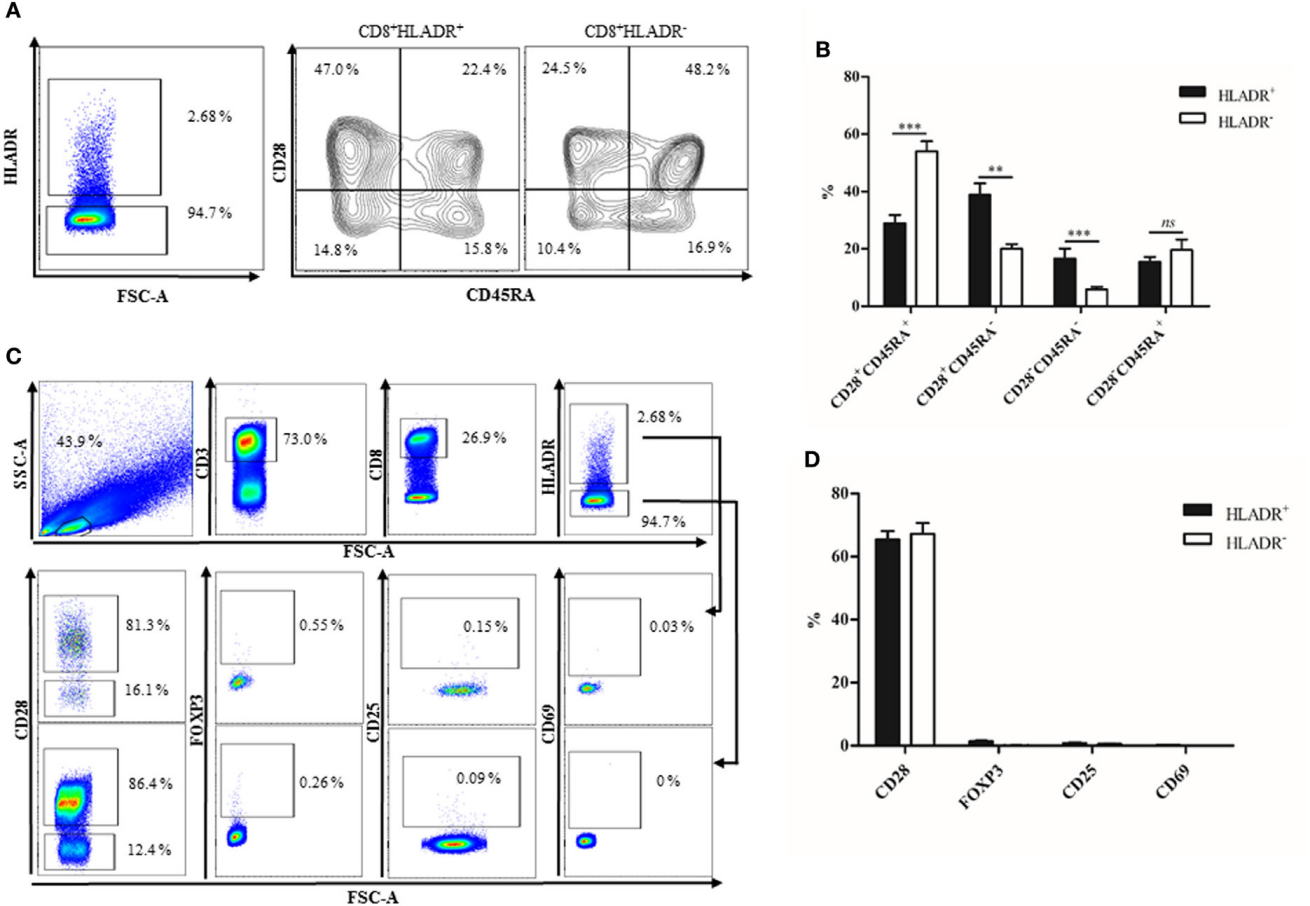
Phenotypical characterization of CD8^+^human leukocyte antigen–antigen D related (HLADR)^+^ and CD8^+^HLADR^−^ T cells. **(A)** Representative FACS plots demonstrating the expression of CD45RA and CD28 in HLADR^+^ and HLADR^−^ cells from a young donor. **(B)** Percentages of HLADR^+^ and HLADR^−^ cells (CD28^+^CD45RA^+^, CD28^+^CD45RA^−^, CD28^−^CD45RA^−^, and CD28^−^CD45RA^+^ cells), young donors *n* = 24 (<30 years), mean ± SEM, **p < 0.001, ***p < 0.0001, ns, not significant. **(C)** Representative FACS plots of activation markers and other molecules in CD8^+^HLADR^+^ and CD8^+^HLADR^−^ cells. The upper panel represents the gating strategy. **(D)** Percentages of CD28, FOXP3, CD25, and CD69 in CD8^+^HLADR^+^ vs CD8^+^HLADR^−^ cells; mean ± SEM, young donors *n* = 22 (<30 years).

The percentage of HLADR^+^ cells within the CD8 T cell fraction was analyzed by FACS (Figure 1), first in a cohort of 91 female Sardinians (Figure 1A) who had previously been recruited and genotyped (1) and second in a young (<30 years) and an elderly (≥70 years) cohort of Austrian males and females (Figure 1B). When HLADR^+^ T cells were correlated with age, there was a highly significant positive correlation in the Sardinian cohort. In the Austrian cohort, CD8^+^HLADR^+^ T cells were compared in young and elderly persons and there was a significant difference between the groups, the elderly persons having a higher percentage of CD8^+^HLADR^+^ T cells. In order to define whether the phenotype of HLADR^+^ T cells corresponded to previous reports (6), FACS analysis of HLADR^+^ cells from young persons was performed (Figure 2). The gating strategy for HLADR^+^ cells and the assessment of CD28 and CD45RA on HLADR^+^ T cells are shown in Figure 2A. The percentages of CD28^+^CD45RA^+^, CD28^+^CD45RA^−^, CD28^−^CD45RA^−^, and CD28^−^CD45RA^+^ cells indicating different differentiation stages from naïve to T_EMRA_-like cells are shown in CD8^+^HLADR^+^ and in CD8^+^HLADR^−^ cells in Figure 2B. Figures 2C,D demonstrate the gating strategy as well as the expression of CD28, FOXP3, CD25, and CD69 in the CD8^+^HLADR^+^ as well as in the CD8^+^HLADR^−^ cell populations. The figures show that CD8^+^HLADR^+^ and CD8^+^HLADR^−^ cells contained cells of every CD8 T cell subset when defined according to the expression of CD28 and CD45RA, although there were fewer cells in the CD28^+^CD45RA^+^ (naïve) subset and more cells in the CD28^+^CD45RA^−^ and the CD28^−^CD45RA^−^ (memory) subsets (Figures 2A,B). In accordance with previous reports (6) neither CD8^+^HLADR^+^ nor CD8^+^HLADR^−^ T cells expressed the classical activation markers CD69 and CD25 and they were negative for FOXP3. The percentage of CD28^+^ cells was identical in the two T cell subsets (Figures 2C,D).

### Expression of Checkpoint Inhibitory Molecules in CD8^+^HLADR^+^ and CD8^+^HLADR^−^ T Cells

**Figure 3:**
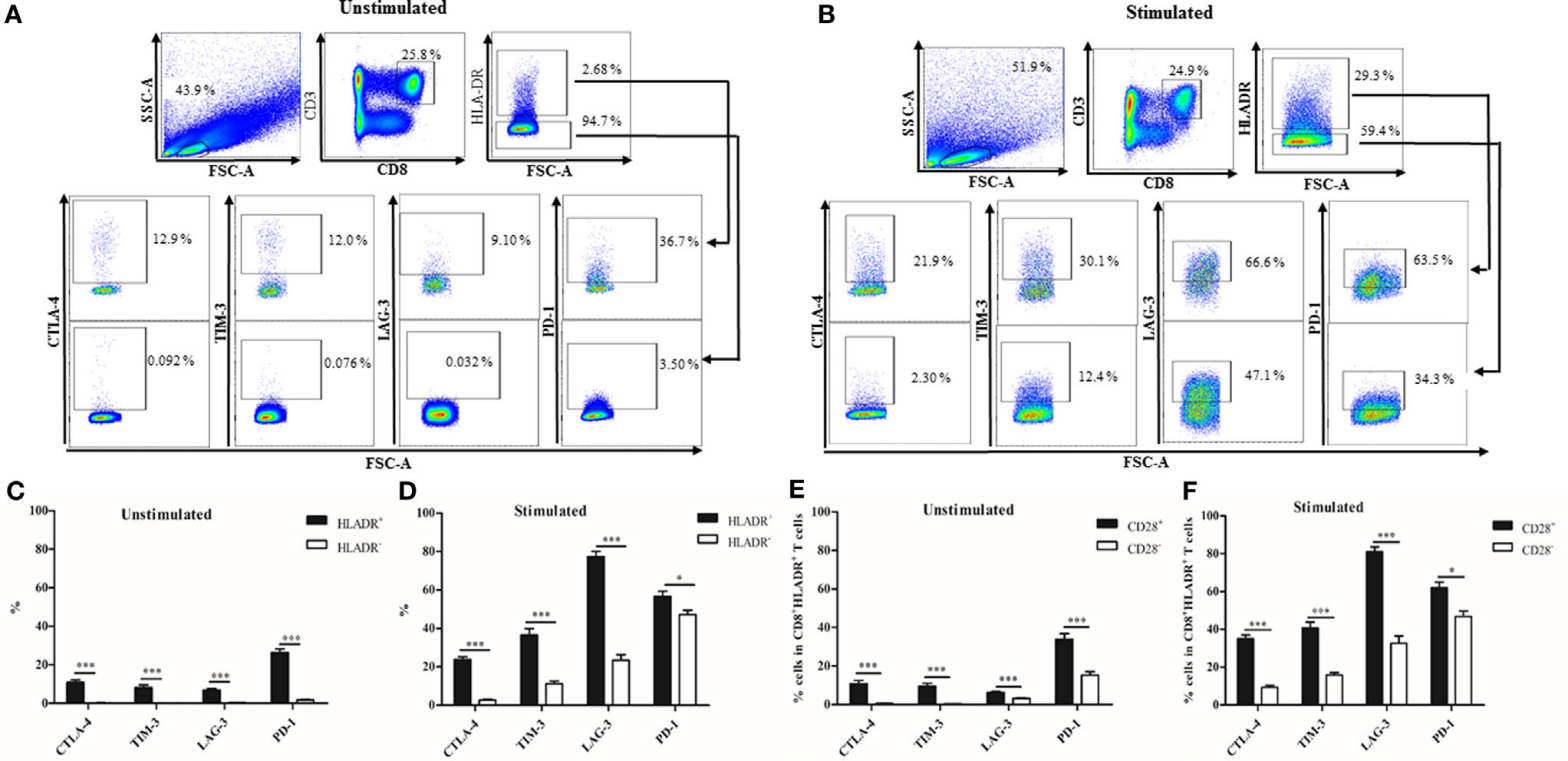
Expression of checkpoint inhibitory molecules on CD8^+^human leukocyte antigen–antigen D related (HLADR)^+^ and on CD8^+^HLADR^−^ T cells. **(A)** Representative FACS plots of inhibitory molecules in the CD8^+^HLADR^+^ (upper panel) and the CD8^+^HLADR^−^ population (lower panel) using cells from a young donor. **(B)** Representative FACS plots of inhibitory molecules in the CD8^+^HLADR^+^ and the CD8^+^HLADR^−^ population using cells from a young donor following stimulation with anti-CD3 (1 µg/ml) and anti-CD28 (0.5 µg/ml) for 24 h. **(C)** Expression of checkpoint inhibitory molecules in CD8^+^HLADR^+^ vs CD8^+^HLADR^−^ T cells in unstimulated cells; young donors *n* = 22 (<30 years), mean ± SEM, ***p < 0.0001. **(D)** Expression of checkpoint inhibitory molecules in CD8^+^HLADR^+^ vs CD8^+^HLADR^−^ T cells following stimulation; young donors *n* = 9 (<30 years), mean ± SEM, ***p < 0.0001, *p < 0.05. **(E)** Expression of checkpoint inhibitory molecules in CD8^+^HLADR^+^CD28^+^ vs CD8^+^HLADR^+^CD28^−^ T cells in unstimulated cells; young donors *n* = 22 (<30 years), mean ± SEM, ***p < 0.0001. **(F)** Expression of checkpoint inhibitory molecules CD8^+^HLADR^+^CD28^+^ vs CD8^+^HLADR^+^CD28^−^ T cells following stimulation; young donors *n* = 9 (<30 years), mean ± SEM, ***p < 0.0001, *p < 0.05.

The checkpoint inhibitory molecules CTLA-4, TIM-3, LAG-3, and PD-1 were analyzed in CD8^+^HLADR^+^ and CD8^+^HLADR^−^ T cells in the resting state (Figures 3A,C). The HLADR^+^ subset contained between 7% (LAG-3) and 28% (PD-1) cells which carried the inhibitory molecules. In contrast, CD8^+^HLADR^−^ cells did not contain checkpoint inhibitory molecules in the resting state. Following stimulation with anti-CD3 and anti-CD28, the percentage of total HLADR^+^ cells expectedly increased (Figure 3B; *p* < 0.0001). The percentage of inhibitory molecule-expressing cells also increased in the stimulated CD8^+^HLADR^+^ as well as in the CD8^+^HLADR^−^ subsets, but there was always a difference between the two populations. CD8^+^HLADR^+^ cells contained more cells expressing inhibitory molecules than CD8^+^HLADR^−^ cells (Figures 3B,D). When one assessed the percentage of cells positive for checkpoint inhibitory molecules in HLADR^+^CD28^+^ and HLADR^+^CD28^-^ T cells, the CD28^+^ subset always contained more cells expressing inhibitory molecules than the CD28^−^ subset (Figure 3E). The same pattern was observed following stimulation of the cells with anti-CD3 and anti-CD28 antibodies (Figure 3F). When we assessed the percentage of cells positive for checkpoint inhibitory molecules in the four populations defined by divergent expression of CD45RA and CD28 (Figure 2) in CD8^+^HLADR^+^ T cells, the CD28^+^CD45RA^+^ population contained more cells expressing CTLA-4, TIM-3, and LAG-3 than the CD28^+^CD45RA^−^, CD28^−^CD45RA^−^, and CD28^−^CD45RA^+^ populations (Figure S1 in Supplementary Material). In contrast, PD1^+^HLADR^+^ cells were more frequent in CD45RA^−^ than in CD45RA^+^ cells. A similar marker profile was observed following stimulation (Figure S1B in Supplementary Material). In CD8^+^ HLADR^−^ cells the expression of checkpoint inhibitory molecules was generally very low even after stimulation (Figures S1C,D in Supplementary Material).

### CD8^+^HLADR^+^ T Cells Have Suppressive Activity Toward the Proliferation of Autologous Stimulated PBMCs

CD8^+^HLADR^+^ T cells have been referred to as “suppressor cells” (6). It was, therefore, our next goal to confirm this assumption in cells from young persons. CD3^+^ cells were purified with magnetic beads and CD8^+^HLADR^+^ T cells were sorted according to a strategy depicted in Figure S2A in Supplementary Material. CD8^+^HLADR^+^ T cells were additionally sub-divided into CD28^+^ and CD28^−^ T cells. The purity of the different fractions was always >95%. The purified subsets were then co-cultured with CFSE- labeled autologous PBMCs and the proliferation of the labeled cells was measured 4 days after stimulation with anti-CD3 and anti-CD28 (Figures S2B–E in Supplementary Material). In the first set of experiments, HLADR^+^ cells were compared with HLADR^−^ cells without considering the expression of CD28 (Figures S2A–D in Supplementary Material). CD8^+^HLADR^+^ T cells suppressed the proliferation of autologous PBMCs in a dose-dependent manner. At a ratio of 4:1 they were still suppressive (Figures S2C,D in Supplementary Material). In Figure S2D in Supplementary Material mean suppressive activities of CD8^+^HLADR^+^ and CD8^+^HLADR^−^ T cells on PBMC proliferation are shown. In spite of a minor not dose-dependent suppressive effect of the CD8^+^HLADR^−^ population, suppression of the CD8^+^HLADR^+^ subset was always higher. In view of the difference in the expression of inhibitory molecules on HLADR^+^CD28^+^ and HLADR^+^CD28^−^ T cells we were interested whether these two subsets had the same suppressive activity. For this reason, the purified HLADR^+^CD28^+^ and HLADR^+^CD28^−^ T cells were added to CFSE-labeled autologous PBMCs. At least at a ratio of 1:2, HLADR^+^CD28^+^ cells had a more pronounced suppressive effect than their CD28^−^ counterparts. At lower cell concentrations there was still a tendency toward increased suppression, but this did not reach statistical significance.

### CD8^+^HLADR^+^ T Cells Occur Also in the BM Where They Have a Similar Phenotype as in the PB

**Figure 4:**
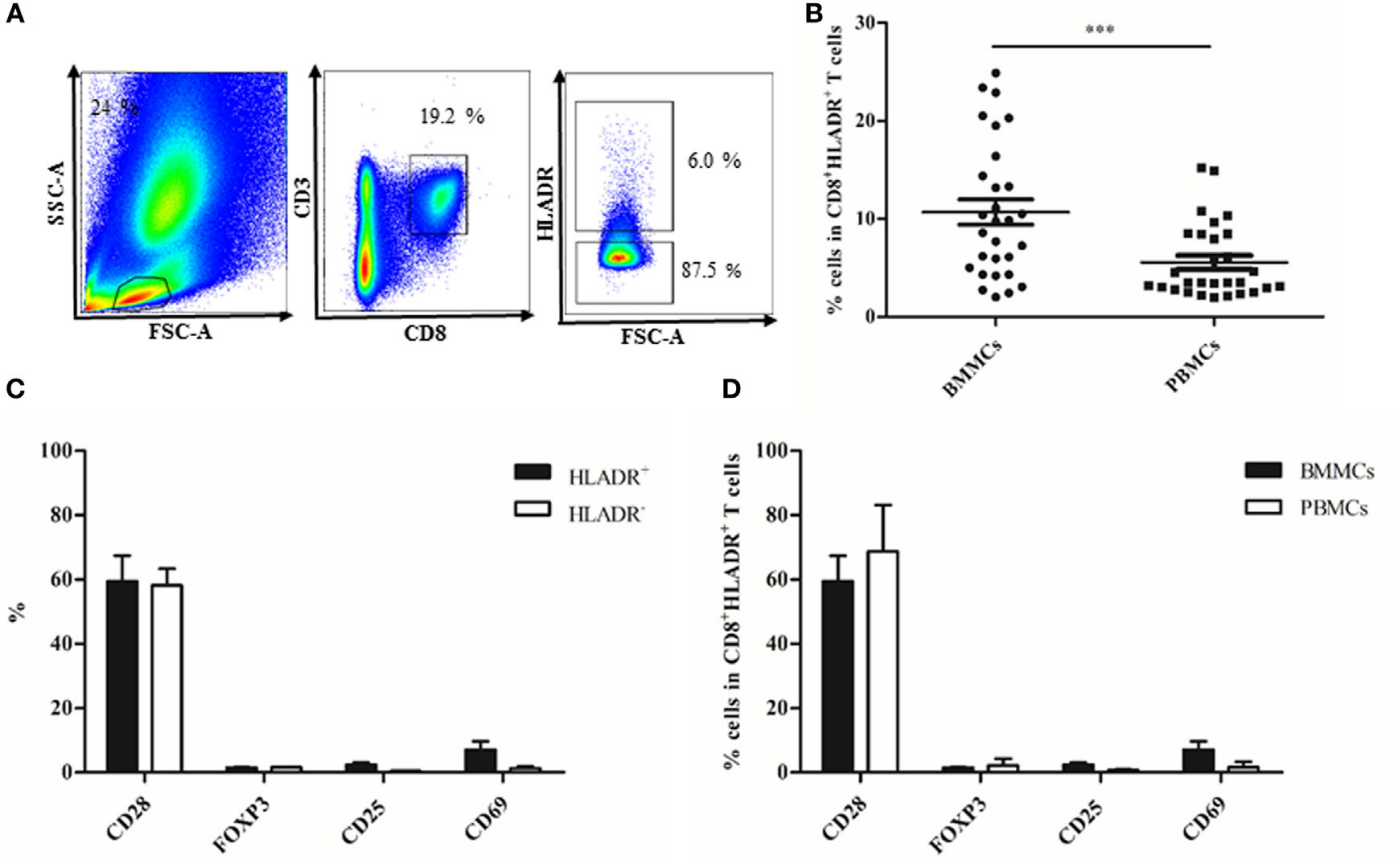
Phenotypical characterization of CD8^+^human leukocyte antigen–antigen D related (HLADR)^+^ and CD8^+^HLADR^−^ T cells in bone marrow mononuclear cells (BMMCs) and peripheral blood mononuclear cells (PBMCs) paired samples. **(A)** Representative FACS plots of the gating strategy in the bone marow. **(B)** Percentages of HLADR^+^ cells in CD8^+^ T cells in BMMCs vs PBMCs; *n* = 29; mean ± SEM, ***p < 0.0001. **(C)** Percentages of CD28, FOXP3, CD25, and CD69 in CD8^+^HLADR^+^ and CD8^+^HLADR^−^ cells in BMMCs; *n* = 5; mean ± SEM. **(D)** Percentages of CD28, FOXP3, CD25, and CD69 in CD8^+^HLADR^+^ cells in BMMCs and PBMCs; *n* = 5; mean ± SEM.

**Figure 5:**
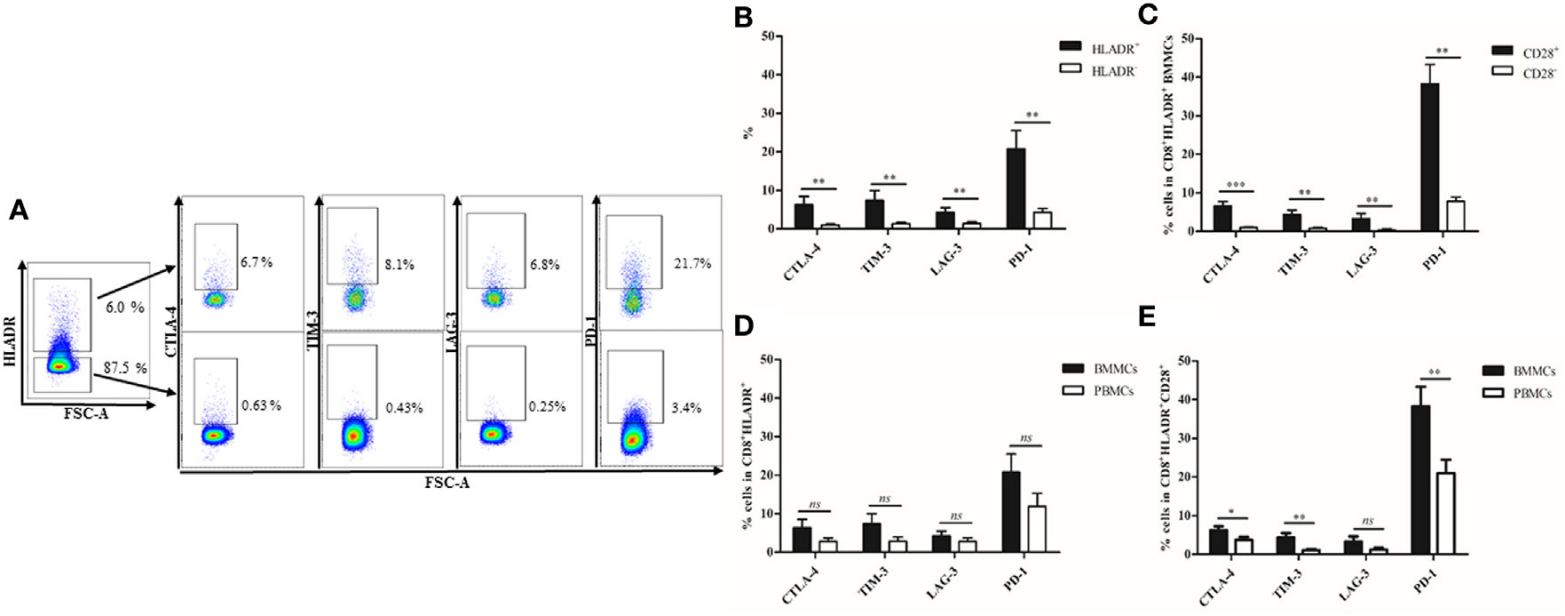
Expression of checkpoint inhibitory molecules on CD8^+^human leukocyte antigen–antigen D related (HLADR)^+^ and on CD8^+^HLADR^+^CD28^+^ T cells in bone marrow mononuclear cells (BMMCs). **(A)** Representative FACS plots of inhibitory molecules in the CD8^+^HLADR^+^ (upper panel) and in CD8^+^HLADR^−^ (lower panel) T cell population in BMMCs. **(B)** Expression of checkpoint inhibitory molecules in CD8^+^HLADR^+^ vs CD8^+^HLADR^−^ cells in BMMCs; *n* = 8; mean ± SEM, **p < 0.001. **(C)** Expression of checkpoint inhibitory molecules in CD8^+^HLADR^+^CD28^+^ vs CD8^+^HLADR^+^CD28^−^ cells in BMMCs; *n* = 8; mean ± SEM, ***p < 0.0001, **p < 0.001. **(D)** Expression of checkpoint inhibitory molecules in CD8^+^HLADR^+^ in BMMCs and in PBMCs; *n* = 8; mean ± SEM, ns, not significant. **(E)** Expression of checkpoint inhibitory molecules in CD8^+^HLADR^+^CD28^+^ in BMMCs and in PBMCs; *n* = 8; mean ± SEM, **p < 0.001 *p < 0.05.

In order to define whether CD8^+^HLADR^+^ T cells occur only in the PB or in other lymphatic organs, we investigated BM samples from the femur of patients of varying age (Table 1) after hip replacement surgery. We and others have recently studied the immunological memory in the human BM in depth (27–31). We were, therefore, interested whether a CD8 regulatory T cell type was also present in this organ. We compared CD8^+^HLADR^+^ T cells in BMMCs and PBMCs (Figure 4) and found that the proportions of this specific cell type were even higher in the BM than in the PB (Figure 4B). As in the PB, BM CD8^+^HLADR^+^ T cells are frequently CD28^+^ and do not express FOXP3 or CD25 (Figure 4C). In contrast to PBMCs, they contain a small proportion of CD69^+^ T cells (Figure 4C), but this is a characteristic feature of BM resting T cells and not of activation (27). The difference between CD8^+^HLADR^+^CD69^+^ T cells in the BM and the PB was also not significant (Figure 4D). As in the PB, cells expressing checkpoint inhibitory molecules were more frequent in the HLADR^+^ than in the HLADR^−^ population and more frequent in HLADR^+^CD28^+^ than in HLADR^+^CD28^−^ cells (Figures 5A–C). When CD8^+^HLADR^+^ T cells were compared in BMMCs and PBMCs there was no significant difference in the number of cells (Figure 5D). However, when only the CD28^+^ population of CD8^+^HLADR^+^ cells was analyzed, checkpoint inhibitory molecules were with the exception of LAG-3 more frequently expressed in BMMCs than in PBMCs (Figure 5E). As the number of samples available from the BM was relatively small and age greatly varied, no correlations with age could be made.

### CD8^+^HLADR^+^ T Cells Have a Lower Expression of Checkpoint Inhibitory Molecules in Old Age

**Figure 6:**
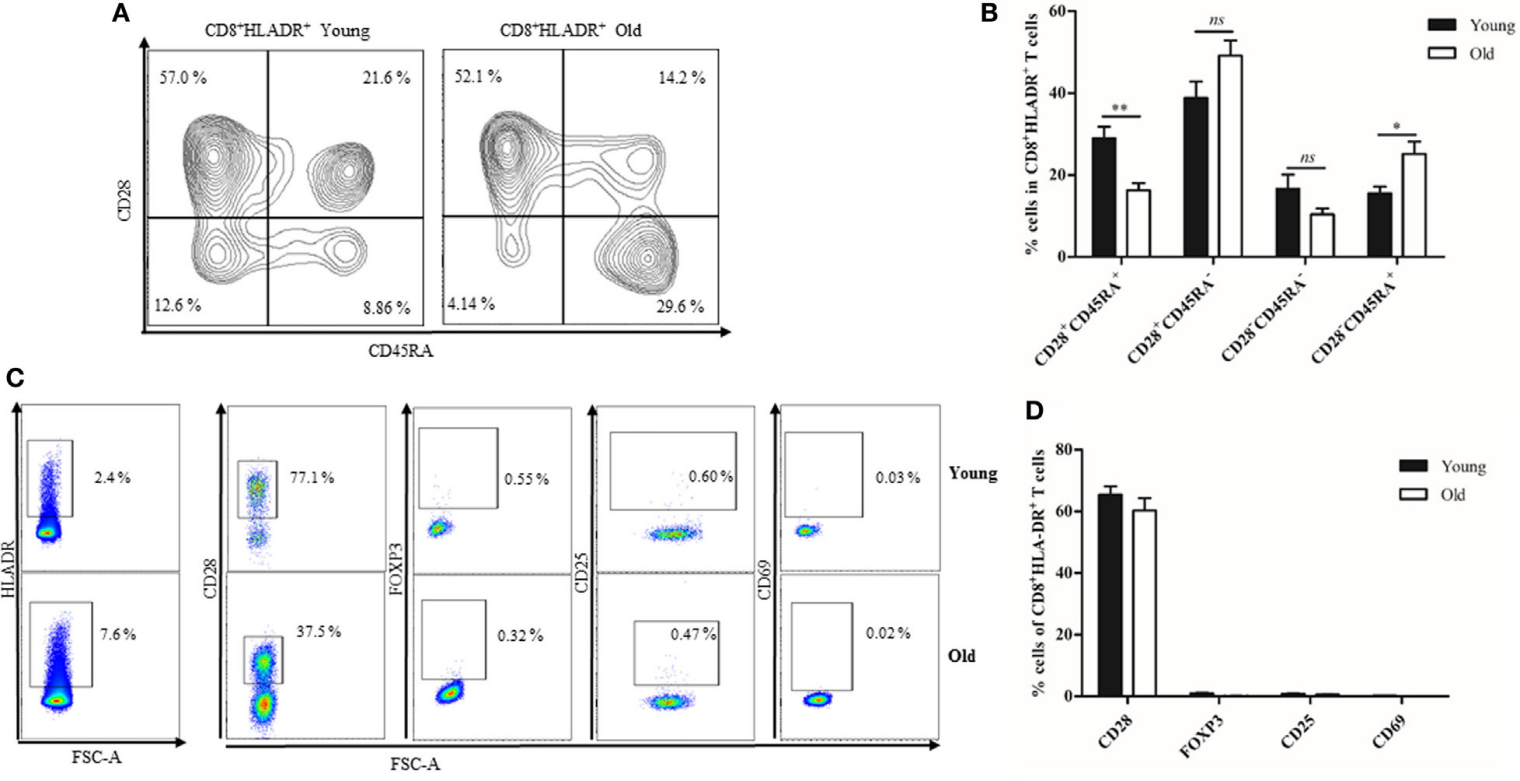
Phenotypical characterization of CD8^+^human leukocyte antigen–antigen D related (HLADR)^+^ T cells from young and old donors. **(A)** Representative FACS plots demonstrating the expression of CD45RA and CD28 in CD8^+^HLADR^+^ cells from a young and an old donor. **(B)** Expression of CD45RA and CD28 in CD8^+^HLADR^+^ cells from young and old donors, CD28^+^CD45RA^+^, CD28^+^CD45RA^−^, CD28^−^CD45RA^−^, CD28^−^CD45RA^+^ cells are shown, young donors *n* = 18–21 (<30 years) and old donors *n* = 18–21 (≥70 years), mean ± SEM, **p < 0.001, *p < 0.05, ns, not significant. **(C)** Representative FACS plots of activation markers and other molecules in CD8^+^HLADR^+^ T cells from a young (upper panel) and an old donor (lower panel). **(D)** Percentages of CD28, FOXP3, CD25, and CD69 cells in the CD8^+^ HLADR^+^ population from young and old donors, young *n* = 18–21 (<30 years) and old donors *n* = 18–21 (≥70 years), mean ± SEM.

We compared CD8^+^HLADR^+^ T cells in the PB from young and elderly persons. Having found that there were more CD8^+^HLADR^+^ T cells in the PB from elderly persons than from young ones (Figure 1), we were now interested to define their phenotype and function. As in samples from young donors, CD8^+^HLADR^+^ T cells were more frequently CD28^+^CD45RA^−^ memory-like T cells than CD28^+^CD45RA^+^ naïve (Figures 6A,B). Decreased percentages of CD28^+^CD45RA^+^ naïve T cells in the CD8^+^HLADR^+^ population combined with increased percentages of CD28^−^CD45RA^+^ T_EMRA_-like cells in the old compared to the young cohort reflected age-related changes typical for the total CD8 T cell pool in old age (26). This suggests that CD8^+^HLADR^+^ T cells do not necessarily represent a separate lineage, but are subject to differentiation such as the CD8^+^HLADR^−^ T cell population. As in the young population, FOXP3, CD25, and CD69 were not expressed in PB CD8^+^HLADR^+^ T cells from elderly persons (Figures 6C,D).

**Figure 7:**
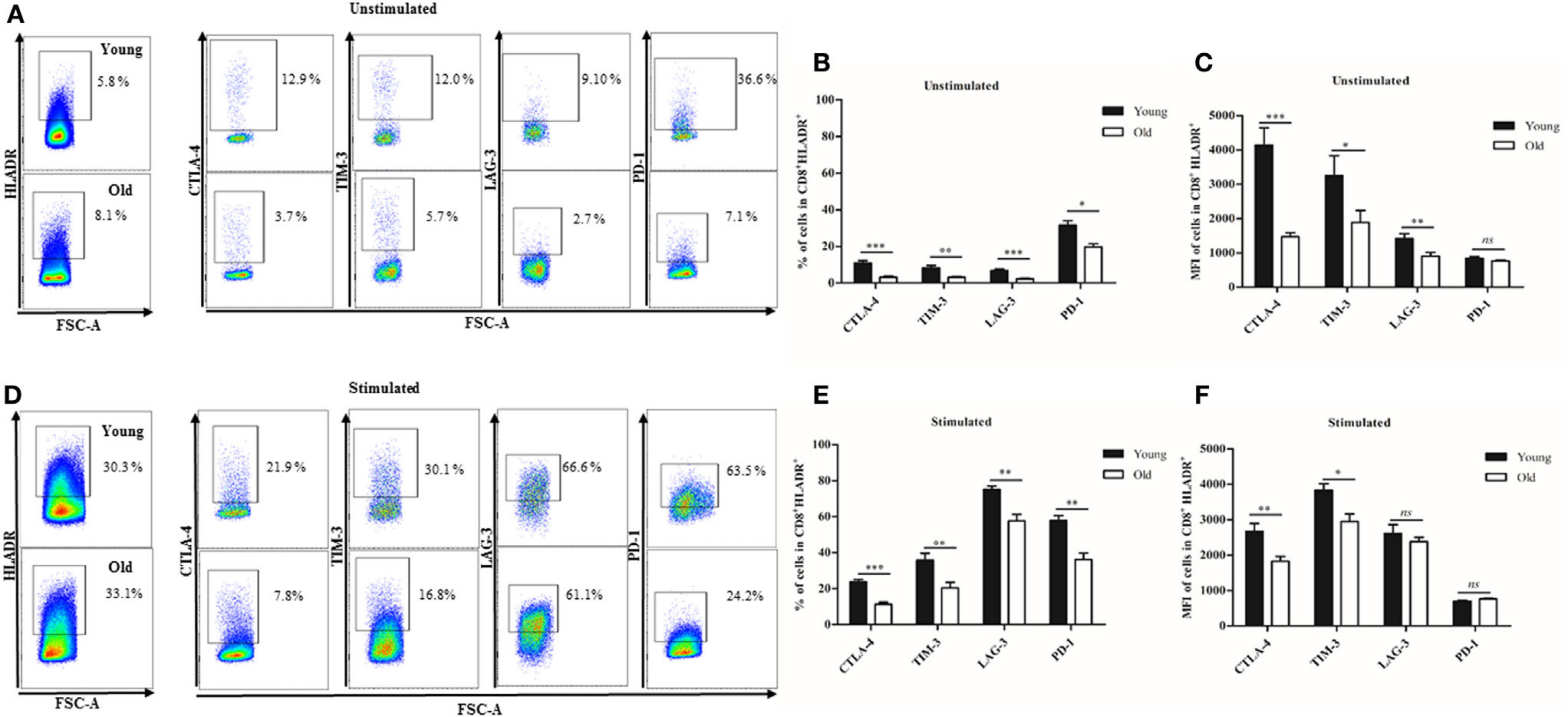
Expression of checkpoint inhibitory molecules on CD8^+^human leukocyte antigen–antigen D related (HLADR)^+^ T cells in young and old persons. **(A)** Representative FACS plots of inhibitory molecules in the CD8^+^HLADR^+^ population from one young (upper panel) and one old (lower panel) donor before stimulation. **(B)** Expression of checkpoint inhibitory molecules (percentages) in CD8^+^HLADR^+^ cells from young and old donors before stimulation; young donors *n* = 22 (<30 years) and old donors *n* = 20 (>70 years), mean ± SEM, ***p < 0.0001, **p < 0.001, *p < 0.05. **(C)** Expression of checkpoint inhibitory molecules (MFI) in CD8^+^HLADR^+^ cells from young and old donors before stimulation; young donors *n* = 20 (<30 years) and old donors *n* = 18 (>70 years), mean ± SEM, ***p < 0.0001, **p < 0.001, *p < 0.05. **(D)** Representative FACS plots of inhibitory molecules in the CD8^+^HLADR^+^ population from one young (upper panel) and one old (lower panel) donor following stimulation with anti-CD3 (1 µg/ml) and anti-CD28 (0.5 µg/ml) for 24 h. **(E)** Expression of checkpoint inhibitory molecules (percentages) in the CD8^+^HLADR^+^ population from young and old donors following stimulation; young donors *n* = 9 (<30 years) and old donors *n* = 7 (>70 years), mean ± SEM, ***p < 0.0001, **p < 0.001. **(F)** Expression of checkpoint inhibitory molecules (MFI) in the CD8^+^HLADR^+^ population from young and old donors; young donors *n* = 9 (<30 years) and old donors *n* = 7 (>70 years), mean ± SEM, ***p < 0.0001, **p < 0.001 *p < 0.05.

When we compared the number of checkpoint inhibitory molecule positive cells in the CD8^+^HLADR^+^ T cell population from young and elderly persons, we found lower percentages of cells expressing CTLA-4^+^, TIM-3^+^, LAG-3^+^, and PD-1^+^ in the old than in the young group (Figures 7A,B). The mean fluorescence intensity of CTLA-4^+^, TIM-3^+^, LAG-3^+^, and PD-1^+^ (MFI) on CD8^+^HLADR^+^ T cells was also lower in the old than in the young group (Figure 7C). This was the case when the total CD8^+^HLADR^+^ population was analyzed (Figures 7B,C) as well as in CD8^+^HLADR^+^CD28^+^ cells (Figures S3A,B in Supplementary Material). The number of checkpoint inhibitory molecule positive cells was low in both groups when cells were unstimulated (Figures 7A–C; Figures S3A,B in Supplementary Material), but increased following stimulation with anti-CD3 and anti-CD28 (Figures 7D–F; Figures S3C,D in Supplementary Material). As in unstimulated cells, the percentage of checkpoint inhibitory positive cells in CD8^+^HLADR^+^ and in CD8^+^HLADR^+^CD28^+^ cells was always higher in the young than in the old cohort following stimulation.

### CD8^+^HLADR^+^ T Cells From Elderly Persons Have Reduced Suppressive Function Toward the Proliferation of Autologous PBMCs

**Figure 8:**
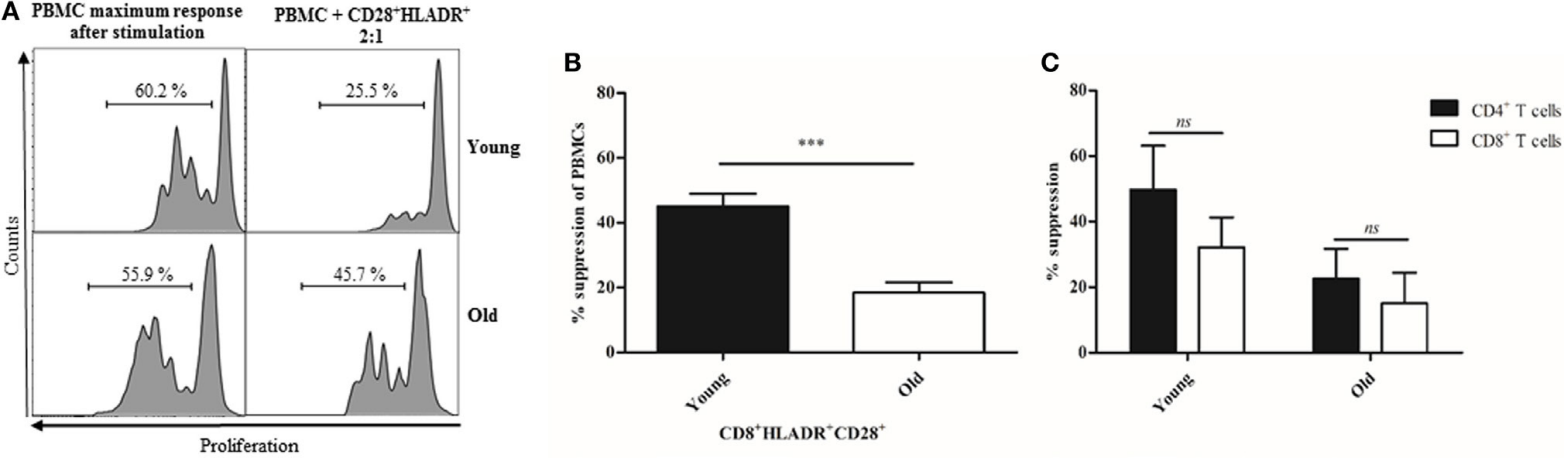
CD8^+^human leukocyte antigen–antigen D related (HLADR)^+^CD28^+^ T cells suppressor properties decrease with age. **(A)** Representative histograms showing the suppressive activity of sorted CD8^+^HLADR^+^CD28^+^ T cells from one young and one old donor on CFSE-labeled peripheral blood mononuclear cells (PBMCs) assessed in co-culture 4 days after stimulation with anti-CD3 (1 µg/ml) and anti-CD28 (0.5 µg/ml). **(B)** Suppressive effect of CD8^+^HLADR^+^CD28^+^ T cells on the proliferation of CFSE-labeled autologous PBMCs (ratio 2:1); mean ± SEM, young donors *n* = 5 (<30 years), old donors *n* = 6 (≥70 years); ***p < 0.001. **(C)** Suppressive effect of CD8^+^HLADR^+^CD28^+^ T cells on the proliferation of CD4^+^ vs CD8^+^ T cells within the CFSE-labeled PBMCs (ratio 2:1); mean ± SEM, young donors *n* = 2 (<30 years), old donors *n* = 4 (≥70 years).

In view of the reduced expression of checkpoint inhibitory molecules on CD8^+^HLADR^+^ T cells in old age, we were interested in the question whether this cell population also had a decreased suppressive function. We, therefore, purified CD8^+^HLADR^+^CD28^+^ T cells from young and elderly persons by cell sorting and co-cultured them with autologous PBMCs labeled with CFSE. In view of low cell numbers available, the co-culture experiment was only performed at a PBMC: HLADR^+^ T cell population ratio of 2:1 (Figure 8). We found that CD8^+^HLADR^+^CD28^+^ T cells from elderly persons had indeed lost most of their suppressive function. Whereas CD8^+^HLADR^+^CD28^+^ T cells from young persons had a suppressive activity of around 40%, suppression was below 20% in cells from elderly persons (Figure 8B). Considering that the number of cells of the different subsets of PBMCs may vary with age, we looked at the proliferation of CD4^+^ and CD8^+^ T cells within the PBMCs. We found no significant differences when we compared the suppression of CD4^+^ T cells vs the suppression of CD8^+^ T cells in both young and old persons (Figure 8C).

### Checkpoint Inhibitory Molecules Mediate the Suppression of CD8^+^HLADR^+^CD28^+^ T Cells

**Figure 9:**
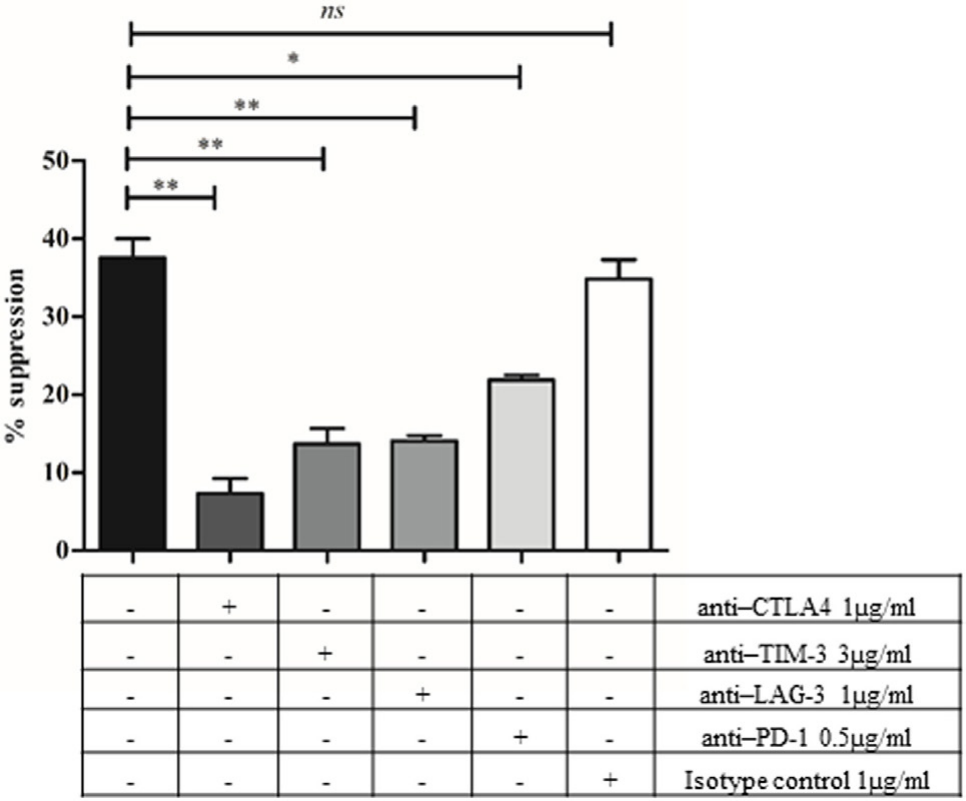
Checkpoint inhibitory molecules are involved in the suppressive activity of CD8^+^human leukocyte antigen–antigen D related (HLADR)^+^CD28^+^ T cells. CFSE-labeled peripheral blood mononuclear cells (PBMCs) were analyzed following a 4 days co-culture after stimulation with anti-CD3 (1 µg/ml) and anti-CD28 (0.5 µg/ml) in the absence or presence of anti-cytotoxic T-lymphocyte-associated antigen 4 (1 µg/ml), anti-T cell immunoglobulin and mucin protein-3 (3 µg/ml), anti-lymphocyte activation gene-3 (1 µg/ml), or anti-programmed death 1 (0.5 µg/ml). Mouse IgG1 isotype Ab was used as control. PBMCs: suppressor cell ratio of 2:1. Young donors *n* = 2–3 (<30 years); mean ± SEM, **p < 0.001, *p < 0.05, ns, not significant.

Checkpoint inhibitory molecules are expressed at a higher level in the CD8^+^HLADR^+^ than in the CD8^+^HLADR^−^ subset. They are also expressed at a higher level in young than in old donors. This may explain the higher suppressive effect of CD8^+^HLADR^+^ T cells of young donors. To check this possibility, we investigated the involvement of these molecules in mediating the suppressive effect of HLADR^+^ cells in neutralizing Ab experiments (Figure 9). We showed that the suppressive effect induced by CD8^+^HLADR^+^CD28^+^ Tregs at a PBMCs: suppressor cell ratio of 2:1 was distinctly inhibited by anti-CTLA-4, anti-TIM-3, anti-LAG-3 and slightly, but still significantly by anti-PD-1. The isotype control had no effect on suppression.

## Discussion

We here show increased numbers of CD8^+^HLADR^+^ T cells in old age in a female Sardinian cohort previously genotyped and described (1) and in a smaller population of young and elderly Austrians. As the number of HLADR^+^ T cells has been demonstrated to be genetically linked (1), it is possible that at least in the Sardinian population the increased numbers of this specific cell type may be driven by the influence of certain genes. However, this is not likely to be the case in the Austrian population. We show here that CD8^+^HLADR^+^ cells most frequently have a CD28^+^ memory phenotype. As elderly persons have more memory than naïve cells (26), it seems plausible that the increased numbers of CD8^+^HLADR^+^ T cells in old age reflect an enlarged CD8^+^ memory T cell pool. In this context, it is also of interest that there are even more CD8^+^HLADR^+^ cells in the BM than in the PB, which may reflect the fact that the BM is known to be a reservoir for memory T cells (27–29, 32). CD8^+^HLADR^+^ T cells have been suggested to represent a separate lineage of T cells, as they occur also in cord blood (6). This possibility cannot be excluded, as we find CD8^+^HLADR^+^ T cells in the naïve cell population in our study. They may still be primed as naïve cells. In consequence they seem to persevere with differentiation, as CD8^+^HLADR^+^ cells occur also in memory as well as effector cell subsets. There is presently no information what the trigger for the induction of a CD8^+^HLADR^+^ phenotype is, but it may on the one hand be antigenic stimulation or also stimulation with cytokines, as suggested by the fact that high numbers are found in the BM, where BM niche cytokines such as IL-7 and IL-15 are prominent (31, 33). Experiments trying to analyze whether *in vitro* stimulation of naïve cells with various BM cytokines can induce this specific phenotype and function are presently underway.

In accordance with previous reports, we demonstrate that CD8^+^HLADR^+^ T cells can inhibit the proliferation of autologous PBMCs and can, therefore, be regarded as Tregs cells (6). As such, they may be an important cell type to maintain homeostatic equilibrium within the immune system. Suppression has previously been suggested to be due to cellular interactions mediated by CTLA-4. We now show that CD8^+^HLADR^+^ cells not only express increased amounts of CTLA-4 but also of other checkpoint inhibitory molecules such as TIM-3, LAG-3, and PD-1. It seems likely that suppression of other cells is not only mediated by one but also by a whole panel of inhibitory molecules. Our results using neutralizing Abs are in favor of this possibility. It was of interest that inhibitory molecules were stronger expressed on the CD28^+^ than the CD28^−^ fraction, which may indicate that pre-stimulation *via* the antigen receptor may be one possible requirement for the induction of inhibitory molecules and their regulatory function.

In this context, it is remarkable that inhibitory molecule expression and regulatory function were decreased in CD8^+^HLADR^+^ T cells from elderly persons in spite of high cell numbers. Decreased T cell receptor signaling is known to be a characteristic feature of old age (34, 35). If inhibitory molecule levels reflect previous antigenic stimulation, checkpoint inhibitory molecule expression would be low in old age as a consequence. In how far high cell numbers could neutralize a decrease in function on a per cell basis is not clear. A similar situation is being discussed for natural killer (NK) cells (36, 37). In the case of CD8^+^HLADR^+^ T cells it seems imaginable that the synergy of a whole panel of different checkpoint inhibitory molecules on the cell surface is needed to trigger the full regulatory capacity of the cells. If these molecules are expressed at low concentrations even after antigenic stimulation, there might be no guarantee that suppressive function is maintained and decreased stimulatory activity would be the consequence.

From our data it is not yet clear toward which cell types the regulatory effect of CD8^+^HLADR^+^ T cells is directed. We can presently only show inhibition of the proliferation of autologous PBMCs as well as CD4^+^ and CD8^+^ T cells. It would be of major interest to define which cell types are target cells of the inhibitory effect and which functions other than proliferation can be influenced. This topic is difficult to study, as purified CD8^+^HLADR^+^ T cells are needed and it is hard to obtain sufficiently high numbers of pure cells after sorting. Isolation of purified cells from the BM is even more difficult. We keep trying to analyze whether BM niche cells (38), monocytes/macrophages, or dendritic cells (DCs) are affected in their stimulatory function. This would influence inflammatory processes. If CD8^+^HLADR^+^ Tregs cells do not function properly in old age, this could then support age-associated subclinical inflammation referred to as “inflamm-aging” (39, 40). As we have recently shown that “inflamm-aging” affects the BM in old age leading to increased levels of oxygen radicals, IL-15, TNF-α, and IFN-γ (31, 33), it is tempting to speculate that lack of regulatory function by CD8^+^HLADR^+^ T cells is a cause of this phenomenon.

In conclusion, we demonstrate for the first time that a decreased expression of checkpoint inhibitory molecules and consecutive lack of suppressive function of the CD8^+^HLADR^+^ T cell type is a characteristic feature of the immune system in old age. It will be of interest to learn more about the functional properties of this specific cell type in regard to inflammation and a possible imbalance between the different types of immune cells.

## Ethics Statement

Cohort A: the study was approved by the Research Ethics and Bioethics Committee of the Consiglio Nazionale delle Ricerche (Italy). Written informed consent was received from participants prior to their inclusion in the study. Cohort B: the study was approved by the Ethics Committees of the Medical University of Innsbruck (Austria). Written informed consent was received from participants prior to their inclusion in the study. Cohort C: the study was approved by the Ethics Committees of the “Klinikum Wels-Grieskirchen” (Austria). Written informed consent was received from participants prior to their inclusion in the study.

## Author Contributions

SY, BG-L, BW, EF, FC, and SS: study design, interpretation of data, critical appraisal, final approval of the version to be published. ML and VO: recruitment of the Sardinian cohort. KT: recruitment of Austrian probands, sample collection, and study design. SY: method design. SS: study design and sorting of cells. SY, MK, FM, and LP: experimental work.

## Conflict of Interest Statement

The authors declare that the research was conducted in the absence of any commercial or financial relationships that could be construed as a potential conflict of interest.

## Abbreviations

BM, bone marrow; BMMCs, bone marrow mononuclear cells; CTLA-4, cytotoxic T-lymphocyte-associated antigen 4; CXCR3, C-X-C motif chemokine receptor 3; FMO, fluorescence minus one; FOXP3, forkhead box protein P3; HLADR, human leukocyte antigen–antigen D related; LAG-3, lymphocyte activation gene-3; PB, peripheral blood; PBMCs, peripheral blood mononuclear cells; PD-1, programmed death 1; TIM-3, T cell immunoglobulin and mucin protein-3; Tregs, regulatory T cells.
