# Supplementary figures and images for: CD8+HLADR+ Regulatory T Cells Change With Aging: They Increase in Number, but Lose Checkpoint Inhibitory Molecules and Suppressive Function
Source: Front Immunol. 2018 Jun 4;9:1201. doi: 10.3389/fimmu.2018.01201 (PMC5994398; doi:10.3389/fimmu.2018.01201)

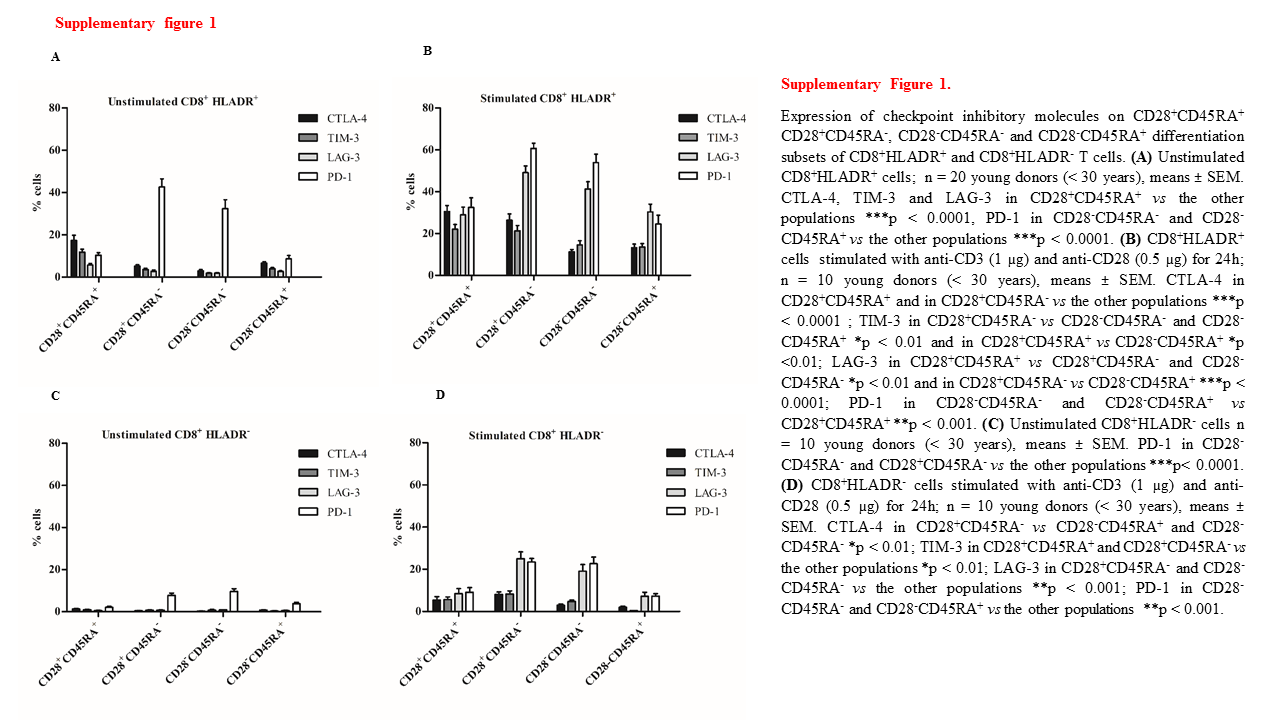

Supplement: Supplementary file 1 [file image_1.tif]

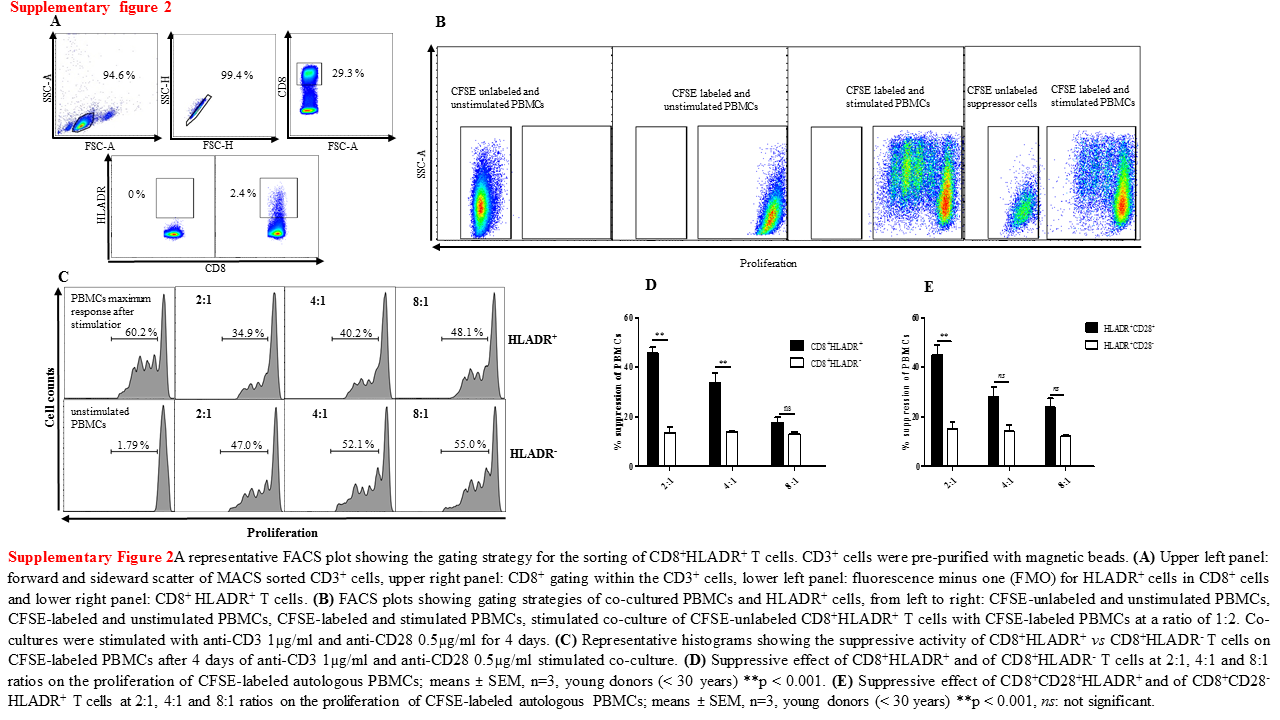

Supplement: Supplementary file 2 [file image_2.tif]

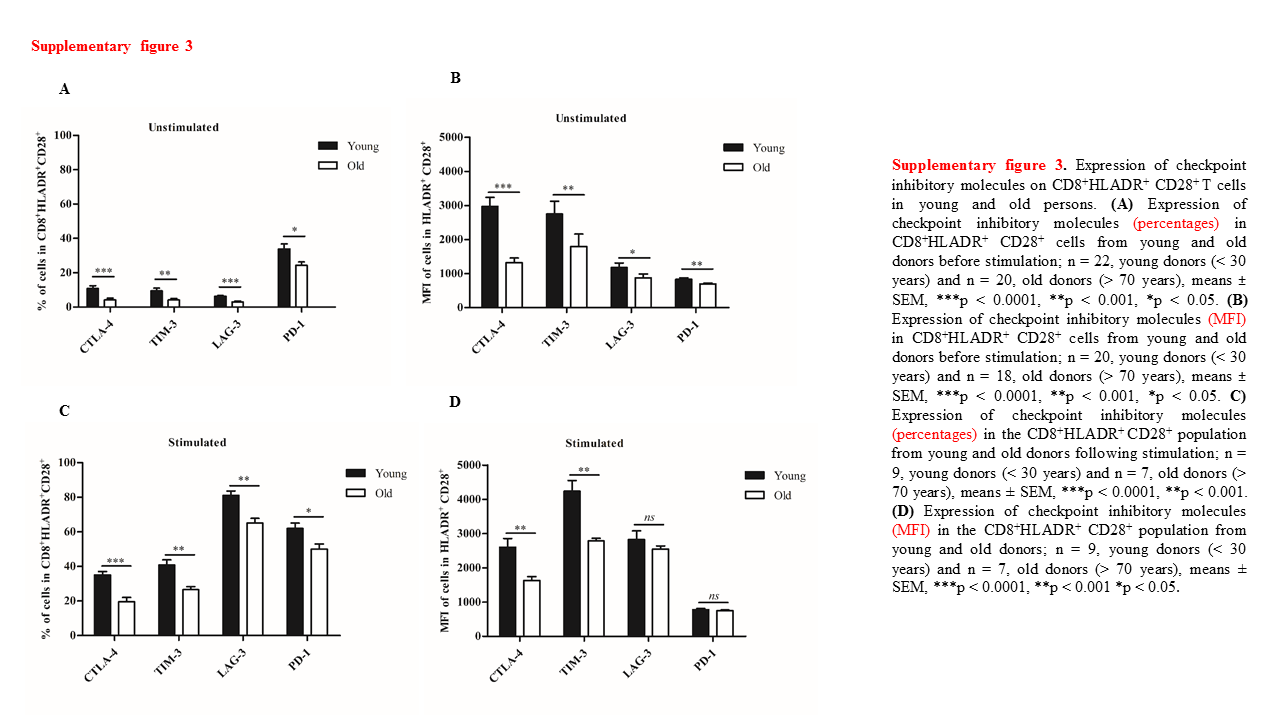

Supplement: Supplementary file 3 [file image_3.tif]
